# Scheduling nab-paclitaxel combined with gemcitabine as first-line treatment for metastatic pancreatic adenocarcinoma

**DOI:** 10.1038/s41416-020-0846-2

**Published:** 2020-04-30

**Authors:** P. G. Corrie, W. Qian, B. Basu, J. W. Valle, S. Falk, C. lwuji, H. Wasan, D. Palmer, M. Scott-Brown, J. Wadsley, S. Arif, J. Bridgewater, D. Propper, R. Gillmore, A. Gopinathan, R. Skells, P. Bundi, R. Brais, K. Dalchau, L. Bax, A. Chhabra, A. Machin, A. Dayim, K. McAdam, S. Cummins, L. Wall, R. Ellis, A. Anthoney, J. Evans, Y. T. Ma, C. Isherwood, A. Neesse, D. Tuveson, D. I. Jodrell

**Affiliations:** 1 Cambridge University Hospitals NHS Foundation Trust (Addenbrooke’s Hospital), Cambridge, UK; 2 Cancer Research UK—Cambridge Institute, University of Cambridge, Cambridge, UK; 3 University of Manchester and The Christie NHS Foundation Trust, Manchester, UK; 4 Bristol Haematology and Oncology Centre, Bristol, UK; 5 Leicester Royal Infirmary, Leicester, UK; 6 Hammersmith Hospital, Imperial College, London, UK; 7 Clatterbridge Cancer Centre, Liverpool, UK; 8 University Hospital Coventry and Warwickshire, Coventry, UK; 9 Weston Park Hospital, Sheffield, UK; 10 Velindre Cancer Centre, Cardiff, UK; 11 UCL Cancer Institute, London, UK; 12 Barts Cancer Institute, London, UK; 13 The Royal Free Hospital, London, UK; 14 Peterborough City Hospital, Peterborough, UK; 15 Royal Surrey County Hospital, Guildford, UK; 16 Western General Hospital, Edinburgh, UK; 17 Royal Cornwall Hospitals, Truro, UK; 18 St. James’s University Hospitals, Leeds, UK; 19 Beatson West of Scotland Cancer Centre, University of Glasgow, Glasgow, UK; 20 Queen Elizabeth Hospital, Birmingham, UK; 21 Gastroenterology and Gastrointestinal Cancer Clinic, University of Göttingen, Göttingen, Germany; 22 Cold Spring Harbor Laboratory, Cold Spring Harbor, New York USA

**Keywords:** Pancreatic cancer, Chemotherapy

## Abstract

**Background:**

Nab-paclitaxel plus gemcitabine (nabP+gemcitabine) offers modest survival gains for patients with metastatic pancreatic ductal adenocarcinoma (PDAC). Sequential scheduling of nabP+gemcitabine in a PDAC mouse model improved efficacy; this hypothesis was tested in a clinical trial.

**Methods:**

Patients with previously untreated metastatic PDAC were randomised to receive nabP+gemcitabine administered either concomitantly on the same day, or sequentially, with gemcitabine administered 24 h after nabP. The primary outcome measure was progression-free survival (PFS). Secondary outcome measures were objective response rate (ORR), overall survival (OS), safety, quality of life (QoL) and predictive biomarkers.

**Results:**

In total, 71 patients received sequential (SEQ) and 75 concomitant (CON) treatment. Six-month PFS was 46% with SEQ and 32% with CON scheduling. Median PFS (5.6 versus 4.0 months, hazard ratio [HR] 0.67, 95% confidence interval [95% CI] 0.47–0.95, *p* = 0.022) and ORR (52% versus 31%, *p* = 0.023) favoured the SEQ arm; median OS was 10.2 versus 8.2 months (HR 0.93, 95% CI 0.65–1.33, *p* = 0.70). CTCAE Grade ≥3 neutropaenia incidence doubled with SEQ therapy but was not detrimental to QoL. Strongly positive tumour epithelial cytidine deaminase (CDA) expression favoured benefit from SEQ therapy (PFS HR 0.31, 95% CI 0.13–0.70).

**Conclusions:**

SEQ delivery of nabP+gemcitabine improved PFS and ORR, with manageable toxicity, but did not significantly improve OS.

**Clinical trial registration:**

ISRCTN71070888; ClinialTrials.gov (NCT03529175).

## Background

Pancreatic ductal adenocarcinoma (PDAC) is the leading cause of cancer mortality.^1^ Without surgery, PDAC is almost uniformly lethal; however, most patients present with unresectable disease. The median overall survival (OS) of patients with metastatic PDAC is under 1 year, even with optimal chemotherapy. The registration MPACT trial established nab-Paclitaxel (nabP) combined with gemcitabine as a standard first-line treatment for patients with metastatic PDAC, reporting improved median OS of 8.7 months for the combination compared with 6.6 months for gemcitabine alone (*p* = 0.001).^2,3^

The mechanism by which nabP enhances gemcitabine efficacy is uncertain. PDAC is a stromal-rich tumour expressing high amounts of secreted protein acidic and rich in cysteine (SPARC). SPARC may act as an albumin-binding protein capable of sequestering nabP to concentrate the drug intra-tumourally. Initial exploration in mice xenografts and PDAC patients treated with nabP indicated drug-induced stromal depletion, which may facilitate gemcitabine delivery to the tumour.^4,5^ However, studies in the KPC genetically engineered mouse model reported that genetic ablation of SPARC did not change intra-tumoural nabP concentrations.^6,7^ In the KPC model, nabP induced reactive oxygen species (ROS) that led to decreases in cytidine deaminase (CDA), a key intracellular enzyme that inactivates gemcitabine. Rapid inactivation of gemcitabine could explain limited antitumour efficacy of gemcitabine in patients with PDAC.^8,9^

The standard treatment schedule of nabP+gemcitabine is to administer gemcitabine to the patient immediately after nabP on days 1, 8 and 15 of a 28-day cycle. Both drugs are given as 30-min infusions. Inhibition of CDA by ROS-mediated degradation demonstrated in the KPC mouse model suggested that sequential administration of nab-paclitaxel and gemcitabine might further increase intra-tumoural gemcitabine concentrations and hence provide a means of enhancing antitumour activity.^6^

The SIEGE (Scheduling nab-paclitaxel with gemcitabine) randomised phase II trial was therefore designed to evaluate whether administration of nabP 24 h in advance of gemcitabine might confer a therapeutic benefit. The MPACT registration trial did not collect quality-of-life (QoL) data from recruited patients, and since SEQ delivery increased the burden of hospital visits for patients, the SIEGE trial included formal evaluation of patient QoL associated with both standard CON and experimental SEQ treatment delivery, using validated questionnaires. To explore the biological rationale for SEQ scheduling in clinical practice, blood and tumour samples were collected from all patients, and both CDA and tumour stromal content were evaluated as potential predictors of treatment outcome.

## Methods

### Patients

Patients aged ≥18 years with histologically-, or cytologically confirmed stage 4 PDAC and previously untreated, measurable metastatic disease, with a Karnofsky performance status (KPS) ≥ 70% were eligible for this trial. Other inclusion criteria included haemoglobin ≥100 g/L, platelets ≥100 × 10^9^/L, white blood cell count ≥3 × 10^9^/L, aspartate aminotransferase and/or alanine aminotransferase ≤2.5 × upper limit of normal (ULN), bilirubin <1.5 × ULN and creatinine ≤1.5 × ULN. The SIEGE trial protocol (ISRCTN71070888) was approved by the Northern Ireland Research Ethics Committee3 and was performed in accordance with the Declaration of Helsinki and the EU Clinical Trials Directive 2001/20/EC. All patients provided written informed consent. Grant funding for this investigator-initiated trial was provided by Celgene Sarl. The funder had no role in study design, data collection, analysis, interpretation or writing of the report.

### Treatment

Patients were randomised 1:1 using a random block method to receive 6 cycles of 4-weekly nabP+gemcitabine, administered either concomitantly (nabP 125 mg/m^2^, 30 min of IV infusion immediately followed by gemcitabine, 1000 mg/m^2^ IV infusion on days 1, 8 and 15 of a 28-day cycle), or sequentially: the same regimen, but gemcitabine administered on days 2, 9 and 16, starting 24 h after commencing nabP. Primary prophylaxis with growth factor (GCSF) support was not mandated. Patients benefiting from treatment could continue beyond 6 cycles at the investigator’s discretion.

### Procedures

Patients were assessed clinically prior to randomisation and at the start of each treatment cycle, then 4-weekly until disease progression and then 3-monthly. Adverse events (AEs) were recorded for up to 30 days after stopping treatment using NCI CTCAE version 4.03. Quality of life (QoL) was assessed using the EORTC QLQ-C30 and QLQ-PAN26 questionnaires at each assessment visit until 12 months. Imaging was performed at baseline and every 8 weeks until disease progression to assess objective response rate (ORR), using RECIST version 1.1. Central radiology retrospective review of RECIST response assessments was planned for 10% of recruited patients. Provision of a pre-treatment tumour biopsy within 12 weeks of randomisation was mandatory. Research blood samples to measure CDA activity were collected pre-randomisation, prior to treatment on day 1 of each cycle, on days 8 and 15 of cycle 1 and at the time of disease progression. Additional samples were collected on days 2, 9 and 16 of cycle 1 for patients allocated sequential therapy.

### Cytidine deaminase (CDA) functional assay

CDA activity was measured in whole blood by a spectrophotometric assay, as previously described.^10^ In brief, CDA releases ammonium during the deamination of cytidine. Therefore, whole-blood samples were diluted 1:10 with water and incubated overnight with cytidine substrate. The concentration of released ammonium was measured using spectrophotometry at 630 nM after coupling with phenol. Protein concentrations were quantified in whole-blood samples diluted 1:200 with water, using the Merck Millipore Direct Detect assay. CDA activity was presented as enzyme activity per milligram of protein (UA/mg).

### Histological assessment of tumour samples

Tumour samples were assessed histologically by a single specialist pathologist on standard H&E sections. Tumour samples were assessed for tumour content, grade and the presence of associated tumour stroma (defined as none, minimal, moderate or extensive). Immunohistochemistry (IHC) for CDA was performed using a commercial polyclonal antibody (Abcam, ad137605) diluted to 1:400. CDA expression was evaluated in both the tumour epithelium and stromal components. For the latter, only stromal spindle cells (representing stromal fibroblasts and myofibroblasts) were evaluated; extracellular constituents such as necrosis, mucin, oedema, haemorrhage and any chronic inflammatory infiltrate were specifically excluded. CDA expression scoring of each of the tumour components (epithelial and stromal cells) were as follows: no staining (negative), focal weak staining (weakly positive), diffuse weak or focal strong staining (positive), diffuse strong staining (strongly positive) or unknown if there was insufficient tumour for assessment.

### Statistical analysis

The primary outcome measure was progression-free survival (PFS). Secondary outcome measures were ORR, OS, safety and QoL. An exploratory analysis was performed to identify potential predictive biomarkers. With 5% significance and 90% power, 55 patients were required to detect a 6-month PFS rate of ≤25% versus ≥44% (or a median PFS of ≤3 months versus ≥5 months) in each arm. Allowing for 25% non-evaluable patients, 146 patients were required. Evaluable patients had to receive at least two cycles of planned treatment with both drugs administered for at least two out of the three planned treatment days in each cycle. Primary analysis was by Kaplan–Meier estimates of 6-month PFS rate and median PFS for each of 2 arms for all patients randomised. The log-rank test was applied to compare PFS and OS between arms; response was compared using the Chi-squared test. The same analysis was repeated for evaluable patients. The subscales of EORTC QLQ-C30 and QLQ-PAN26 questionnaires were derived according to standard-scoring manuals, and the scores were standardised to a range of 0–100. Clinically important differences were categorised as small if the mean score change was 5–10 points, moderate for 10–20 points and large for >20 points.^11^ Analyses of changes from baseline over time and difference between the 2 arms for subscales were carried out with repeated measures using ANCOVA, adjusting for baseline level.

Biomarker outcomes were analysed in an exploratory manner. The Pearson and Spearman correlation coefficient, Wilcoxon/Kruskal–Wallis and regression models were applied to explore the correlation between pre-treatment CDA activity and patient characteristics (age, gender, KPS, site of primary tumour, liver metastases, serum carbohydrate antigen 19.9 (CA19.9), C-reactive protein (CRP) and neutrophil count). Descriptive statistics for CDA activity in all samples associated with cycle 1 in both arms were undertaken and depicted graphically to assess the impact of CDA activity on CON versus SEQ scheduling. CDA activity in both arms was measured on cycle 1 day 1, day 8, day 15 as well as on day 1 of cycles 2, 4 and 6 and compared using the Wilcoxon test. In the SEQ arm, differences in CDA activity measured in cycle 1 on days 1 and 2, days 8 and 9 and days 15 and 16 were evaluated using the Wilcoxon test to establish changes after 24-h exposure to nabP. Boxplots of pre-treatment CDA by ORR and CTCAE grade were generated and the Kruskal–Wallis test applied. Cox regression models were applied to assess the predictive value of CDA for PFS and OS.

Distributions and frequencies of tumour grade, CDA expression and stroma content by IHC, and their correlation with PFS and OS, were analysed for each of the treatment arms, as well as for all study patients. Kaplan–Meier plots were generated for each of the treatment arms and for all study patients, by tumour grade, CDA expression and stroma content. Hazard ratios were estimated using Cox regression models.

## Results

### Patients and treatment delivery

From March 2014 to March 2016, 146 patients (75 concomitant [CON] arm, 71 sequential [SEQ] arm) were recruited at 19 UK centres (Trial Flow Diagram is provided in Supplementary Fig. S1). Patient demographics and pre-treatment disease characteristics were well balanced between the two arms (Table 1). Median patient age was 66 years, 43% were female and 62% had KPS > 90%.

**Table 1:** Patient pre-treatment characteristics.

	CON (*n* = 75)	SEQ (*n* = 71)
*Age*		
Median (range)	67 (48, 82)	63 (45, 77)
*Gender*		
Male	40 (53%)	43 (61%)
Female	35 (47%)	28 (39%)
*Karnofsky performance status*		
100	17 (23%)	18 (25%)
90	33 (44%)	22 (31%)
80	19 (25%)	20 (28%)
70	6 (8%)	11 (15%)
*ECOG performance status*		
0	33 (44%)	30 (43%)
1	38 (51%)	39 (56%)
2	4 (5%)	1 (1%)
Unknown	0	1
*Site of primary tumour*		
Head	35 (47%)	34 (48%)
Body	19 (25%)	20 (28%)
Tail	21 (28%)	17 (24%)
*Liver metastases*		
No	13 (17%)	11 (15%)
Yes	62 (83%)	60 (85%)
*CA19.9 (U/ml)*		
Median (range)	4032 (1.0, 275228)	2377 (4.0, 217920)
<ULN	9 (13%)	9 (13%)
≥ULN	59 (87%)	60 (87%)
Unknown	7	2
*C-reactive protein (mg/l)*		
Median (range)	9.0 (0.8, 249)	11.0 (0.3, 208)
Unknown	5	1
*Neutrophil count (10*^*9*^*/l)*		
Median (range)	5.3 (1.6, 19.3)	6.1 (2.6, 22.4)

The median (range) number of nabP+gemcitabine chemotherapy cycles received was 3 (0–12) in the CON arm and 4 (0–24) in the SEQ arm. In the SEQ arm, a 24 + /− 2-h interval between nabP and gemcitabine administration was achieved in 94% of 828 treatment administrations. Twenty-one (28%) patients in the CON and 30 (42%) patients in the SEQ arm completed ≥6 cycles of planned treatment. The most common reasons for patients stopping treatment sooner were AEs (20 CON versus 22 SEQ arm patients) and progressive disease (23 CON versus 11 SEQ arm patients). Thirty-eight (26%) patients received second-line chemotherapy (22 CON versus 16 SEQ arm patients).

### Toxicity

Four patients who were randomised did not start chemotherapy: 1 CON arm patient progressed rapidly and died within 1 month of enrolment; 2 SEQ arm patients withdrew consent; 1 SEQ arm patient was subsequently found to be ineligible. Therefore, safety analysis was based on 142 patients. The incidence of any AE in both arms was similar, but severity was higher overall in the SEQ arm (Table 2). Grade ≥3 AEs were reported in 61 (82%) patients (average 0.69/cycle) in the CON arm, but in 66 (97%) patients (average 0.87/cycle) in the SEQ arm. SEQ treatment was associated with a higher incidence of grade ≥3 neutropaenia (24 CON versus 40 SEQ arm), although febrile neutropaenia rates were similar (9 CON versus 11 SEQ arm). GCSF was administered to 12 (16%) patients in the CON arm and 24 (35%) patients in the SEQ arm as secondary prophylaxis.

**Table 2:** Summary of Grade > 3 CTCAEs occurring in at least 5% patients.

	CON (*n* = 74)						SEQ (*n* = 68)					
	Grade 3,		Grade 4,		Grade 5,		Grade 3,		Grade 4,		Grade 5,	
	*n*	%	*n*	%	*n*	%	*n*	%	*n*	%	*n*	%
No. of patients experiencing worst-grade AEs	44	59	12	16	5	7	29	43	27	40	10	15
Fatigue	15	20	0	0	0	0	16	24	0	0	0	0
Neutrophil count decreased	20	27	4	5	0	0	19	28	21	31	0	0
Nausea	2	3	0	0	0	0	6	9	0	0	0	0
Diarrhoea	4	5	0	0	0	0	6	9	0	0	0	0
Anaemia	4	5	0	0	0	0	8	12	0	0	0	0
Vomiting	8	11	0	0	0	0	5	7	0	0	0	0
Abdominal pain	4	5	0	0	0	0	3	4	0	0	0	0
Platelet count decreased	7	9	1	1	0	0	2	3	1	1	0	0
White blood cell decreased	1	1	2	3	0	0	4	6	2	3	0	0
Lung infection	4	5	0	0	3	4	4	6	0	0	2	3
Febrile neutropaenia	6	8	2	3	1	1	7	10	3	4	1	1
GGT increased	5	7	2	3	0	0	2	3	1	1	0	0
Alanine aminotransferase increased	2	3	0	0	0	0	5	7	1	1	0	0
Thromboembolic event	2	3	0	0	0	0	4	6	0	0	1	1
Bilirubin increased	1	1	0	0	0	0	8	12	0	0	0	0
Sepsis	0	0	5	7	0	0	0	0	4	6	2	3
Dehydration	3	4	0	0	0	0	5	7	0	0	0	0
Hypotension	3	4	0	0	0	0	4	6	0	0	0	0
Other grade 5 AEs	1 gallbladder obstruction						1 pneumonitis, 1 colitis, 1 hypoxia and 1 cholangitis					

The dose intensity of both nabP and gemcitabine was 17% lower in the SEQ arm: the median dose per 4-week cycle in the CON and SEQ arms, respectively, was 300 mg/m^2^ versus 249 mg/m^2^ for nabP and 2400 mg/m^2^ versus 2000 mg/m^2^ for gemcitabine. The total cumulative dose of both nabP and gemcitabine was, however, higher in the SEQ arm: median total dose per patient for nabP and gemcitabine was 750 mg/m^2^ and 6290 mg/m^2^ in the CON arm and 984 mg/m^2^ and 7470 mg/m^2^ in the SEQ arm, consistent with longer treatment duration with SEQ delivery. Rates of radiologically confirmed pneumonitis were low overall: 3 patients in each arm. Rates of peripheral neuropathy were similar (22 CON and 22 SEQ arm patients) and grade 1–2 in all cases, except 5 patients who experienced grade 3 events (2 CON and 3 SEQ arm). Seven deaths were attributed to treatment: 3 CON arm (2 pneumonia and 1 febrile neutropaenia) and 4 SEQ arm (1 pneumonitis, 1 colitis, 1 hypoxia and 1 febrile neutropaenia).

### Efficacy

Local assessment of radiological response was undertaken in 117 (80% of total recruited) patients: 61 in the CON and 56 in the SEQ arm. Central radiology review of local RECIST assessment was performed in 11 randomly selected patients for whom at least 1 response assessment was performed and showed high concordance in assessment of measurable target lesions. ORR was higher with SEQ therapy: 29/56 (52%) versus 19/61 (31%) evaluable patients experienced a complete or partial response (*p* = 0.023, Supplementary Table S1).

**Fig. 1:**
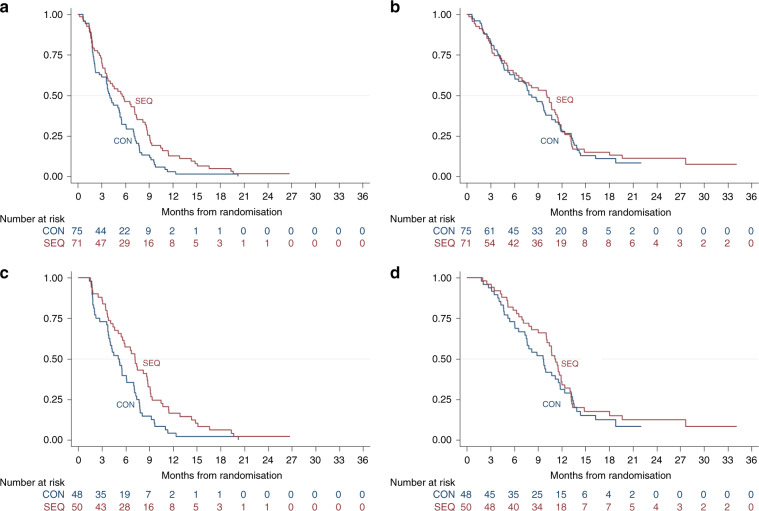
Kaplan-Meier curves of progression-free survival (PFS) and overall survival (OS). **a**, **c** PFS and (**b**, **d**) OS for intention-to treat (**a**, **b**) and evaluable (**c**, **d**) patients.

With a median follow-up of 21.4 months, 133 (70 CON and 63 SEQ arm) patients had progressed or died, and 123 (64 CON and 59 SEQ arm) patients had died. The 6-month and median PFS were 32% (95% CI 21–43%) and 4.0 months (95% CI 3.0–5.4 months) in the CON arm and 46% (95% CI 34–58%) and 5.6 months (95% CI 3.6–7.2 months) in the SEQ arm (HR 0.67 favouring the SEQ schedule, 95% CI 0.47–0.95, *p* = 0.022) (Fig. 1a). The median OS was 8.2 months (95% CI 6.1–10.0 months) in the CON arm versus 10.2 months (95% CI 6.3–11.5 months) in the SEQ arm (HR 0.93, 95% CI 0.65–1.33, *p* = 0.70) (Fig. 1b). For evaluable patients, survival benefits with SEQ therapy appeared greater, although OS still did not meet statistical significance: PFS HR 0.60 (95% CI 0.40–0.90, *p* = 0.014) and OS HR 0.83 (95% CI 0.54–1.28, *p* = 0.40) (Fig. 1c, d; Supplementary Table S2).

### Quality of life

**Fig. 2:**
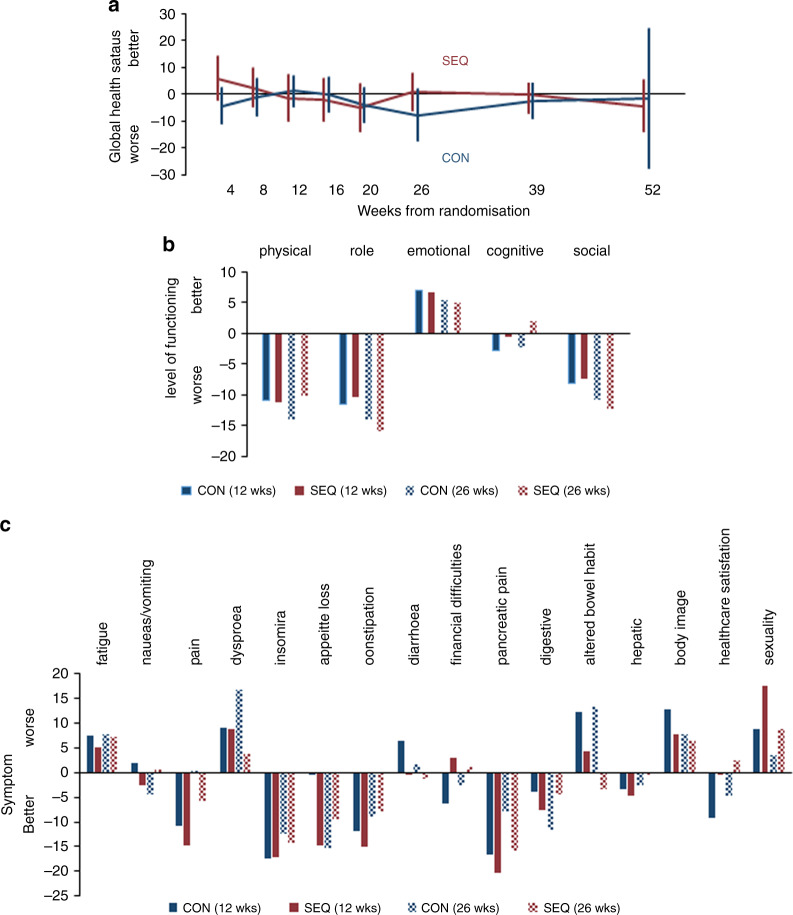
Changes from baseline in EORTC QLQ-C30 QoL scores associated with treatment. **a** Global health status (**b**) QoL subscales (**c**) symptoms.

A total of 523 QoL questionnaires were returned by 141 (97%) patients (72 CON and 69 SEQ arm) during the course of this trial: 138 (95%) recruited patients (70 CON and 68 SEQ arm) completed baseline EORTC QLQ-C30 and PAN26 questionnaires. The number of questionnaires returned fell over time, as expected, although returns were consistently higher in the SEQ arm: 79 (39 CON and 40 SEQ) at 12 weeks, 59 (26 CON and 33 SEQ) at 26 weeks, 45 (17 CON and 28 SEQ) at 39 weeks and 17 (5 CON and 12 SEQ) at 52 weeks. The mean pre-treatment global health status score from the QLQ-C30 questionnaire on a scale of 0–100 was 62 (63 for the CON and 61 for the SEQ arm). Global health status scores were overall stable over time (i.e. changes from baseline were less than 5 points) and similar in both arms, with the exception of a small deterioration of >5 points in the CON arm at 26 weeks (Fig. 2a).

Since relatively few QoL assessments were available at later time points, comparisons between the two arms mainly focused on the first 26 weeks. The mean scores at baseline and changes from baseline at 12 and 26 weeks of all QoL subscales derived from the EORTC QLQ-C30 and PAN26 questionnaires were similar for the two arms. For both trial arms, physical, role and social functioning recorded by the QLQ-C30 questionnaire worsened over time, while emotional functioning showed slight improvement (Fig. 2b). For specific symptoms (Fig. 2c), treatment appeared to improve pain, pancreatic pain, insomnia, appetite and constipation. The gains were generally less evident at 26 weeks compared with 12 weeks. Treatment was associated with an increase in breathlessness, altered bowel habit and to a lesser extent worsening perceptions of body image and sexuality. Again, all these symptoms were less apparent at 26 weeks compared with 12 weeks. There were 2 statistically significant differences from baseline levels identified between the 2 trial arms, both favouring SEQ therapy: better appetite at 12 weeks (*p* = 0.047) and less change in bowel habit at 26 weeks (*p* = 0.003). Breathlessness at week 26 was reported to be more severe in the CON arm, although the difference between the two arms did not reach statistical significance (*p* = 0.065). More detailed breakdown of QoL data is provided in Supplementary Table S3.

### Whole-blood CDA activity

Pre-treatment whole-blood CDA activity (measured pre- and repeated post randomisation), with time intervals between samples ranging 0–26 days, showed similar mean (14.0 and 14.3 UA/mg, respectively) and median (11.5 and 11.9 UA/mg, respectively) values, as well as low intra-patient variability (coefficient of variation = 18%). For subsequent analyses, the mean value of pre-treatment CDA activity measurements was used as the baseline CDA activity.

**Fig. 3:**
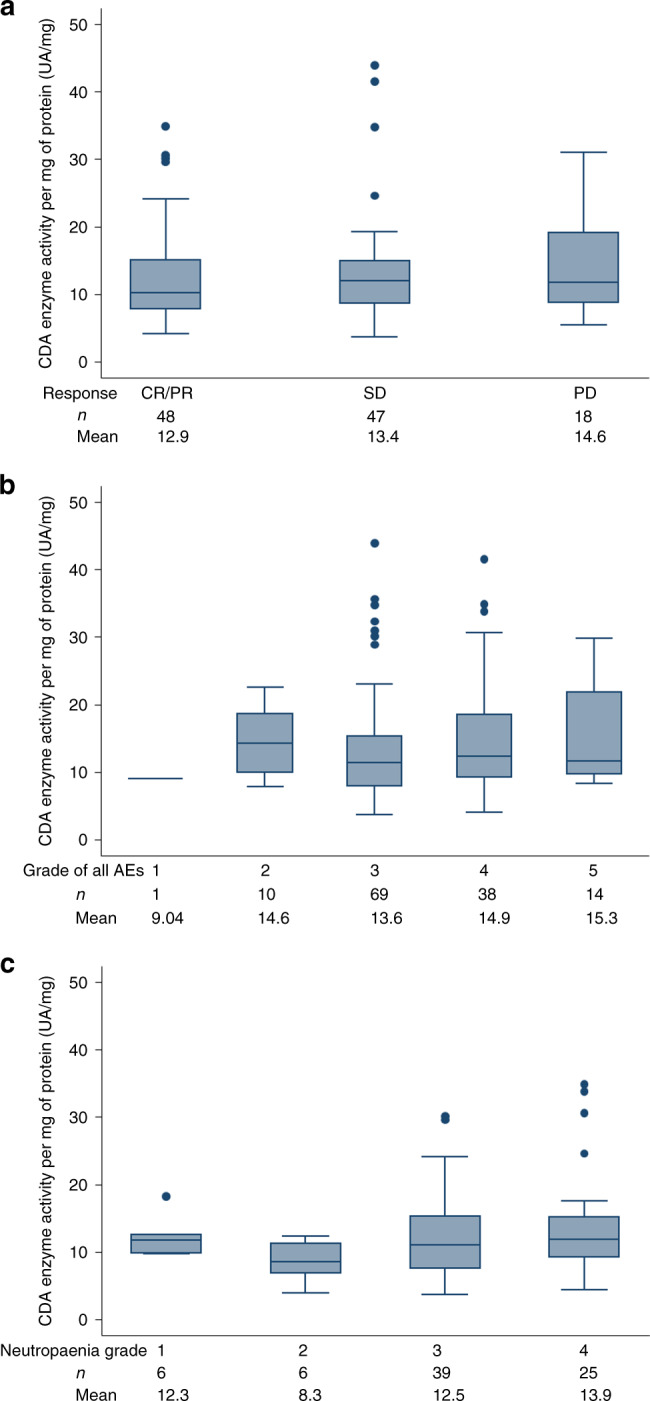
Box-plots demonstrating that baseline (pre-treatment) cytidine deaminase (CDA) activity did not predict for objective response or toxicity experienced by individual patients during treatment. **a** objective response (*p* of Kruskal–Wallis test = 0.62), (**b**) worst CTCAE grade experienced (*p* of Kruskal–Wallis test = 0.63) and (**c**) worst CTCAE neutropaenia grade experienced (*p* of Kruskal–Wallis test = 0.38).

Baseline whole-blood CDA activity correlated with neutrophil count for the whole-trial population (correlation coefficient 0.82, *p* < 0.0001, Supplementary Table S4, Supplementary Fig. S2) after adjusting for other baseline patient characteristics, but did not predict for ORR (Fig. 3a), grade of treatment toxicity (Fig. 3b) or grade of neutropaenia experienced (Fig. 3c). Neither was there any difference in pre-treatment whole-blood CDA activity between those patients who experienced grade >3 haematological toxicity and who did not either during the first cycle, or during the first two cycles (Supplementary Fig. S3). After adjusting for pre-treatment neutrophil count, baseline CDA activity did not correlate with PFS or OS (Supplementary Table S5).

During the first cycle, CDA activity decreased incrementally from baseline levels in both trial arms, with significantly greater reduction at day 8 in the SEQ arm compared with the CON arm: 77% versus 47%, *p* < 0.0001, but this was no longer seen in the CON arm after adjusting for neutrophil count (Supplementary Fig. S4). However, the mean reduction from pre-treatment values on day 15 was similar in both arms: 59% SEQ and 65% CON; moreover, subsequent CDA activity measured on day 1, cycle 2 and during further cycles was similar to baseline levels for both patient groups.

### Tumour CDA and stroma expression

Sufficient tumour tissue from 128 patients (65 CON and 63 SEQ arm) was available for IHC analysis. Tumour grade, CDA expression and stroma content and their correlation with PFS and OS associated with chemotherapy treatment are summarised in Table 3 and Supplementary Fig. S5. When comparing the SEQ with the CON treatment arm, strongly positive tumour epithelial CDA staining was evident in 34 tumours and appeared to predict for improved PFS, but not OS associated with SEQ therapy (HR 0.31, 95% CI 0.13–0.70). Eight patients whose tumours had strongly positive CDA expression received second-line chemotherapy on disease progression: 6 patients in the CON arm and 2 patients in the SEQ arm. In this small-size cohort, there was a trend towards strongly positive CDA expression predicting for longer OS with SEQ therapy (HR 0.74, 95% CI 0.36–1.56). Patients with strong tumour CDA expression had higher whole-blood CDA activity compared with patients with lower levels of staining (*p* = 0.031, Supplementary Table S6).

**Table 3:** Summary of tumour sample immunohistochemical analyses and correlation with PFS and OS.

	CON (*n* = 65)	SEQ (*n* = 63)	HR, 95% CI for PFS	HR, 95% CI for OS
*Tumour grade*				
Well differentiated/moderately differentiated	2 (4%)/38 (67%)	2 (4%)/38 (72%)	0.65, 0.41–1.04	0.86, 0.53–1.39
Poorly differentiated	17 (30%)	13 (24%)	0.77, 0.35–1.70	1.08, 0.50–2.33
Unknown^a^	8	10		
Prognostic: p log rank = 0.41 (PFS), 0.12 (OS)				
*CDA expression—tumour epithelium*				
Negative/weakly positive/positive	3 (6%)/8 (15%)/ 25 (47%)	0/10 (19%)/25 (48%)	0.91, 0.56–1.48	1.07, 0.65–1.76
Strongly positive	17 (32%)	17 (33%)	0.31, 0.13–0.70	0.74, 0.36–1.56
Unknown^a^	12	11		
Prognostic: p log rank = 0.24 (PFS), 0.88 (OS)				
*CDA expression—stromal cells*				
Weakly positive/positive	14 (34%)/22 (54%)	17 (40%)/21 (50%)	0.74, 0.46–1.20	1.10, 0.67–1.79
Strongly positive	5 (12%)	4 (10%)	0.73, 0.17–3.07	1.49, 0.33–6.81
Unknown^a^	24	21		
Prognostic: p log rank = 0.12 (PFS), 0.48 (OS)				
*Presence of tumour stroma*				
None/minimal	11 (19%)/14 (24%)	7 (13%)/24 (45%)	0.56, 0.30–1.03	0.79, 0.45–1.38
Moderate/extensive	19 (33%)/14 (24%)	13 (24%)/9 (17%)	0.60, 0.34–1.06	0.85, 0.47–1.53
Unknown^a^	7	10		
Prognostic: p log rank = 0.027 (PFS), 0.023 (OS)				

^a^Unknown = insufficient tumour sample to analyse.

Baseline tumour stromal content did not differ between the 2 trial arms. Patients whose tumours had moderate or extensive tumour stroma staining had longer PFS and OS on nabP+gemcitabine, irrespective of the trial arm. The unadjusted and adjusted (for baseline characteristics of age, gender, KPS, location of primary tumour in the pancreas, liver metastases, number of target lesions reported, CA19.9, CRP and ANC) PFS HRs were 0.64 (95% CI 0.43–0.95) and 0.65 (95% CI 0.42–1.01), while the OS HRs were 0.63 (95% CI 0.42–0.94) and 0.52 (95% CI 0.32–0.83), respectively.

## Discussion

In this first, prospective randomised trial exploring alternative scheduling of nabP with gemcitabine, SEQ delivery of nabP+gemcitabine significantly improved PFS compared with standard CON delivery, meeting the trial primary endpoint. However, this did not translate into an OS benefit. Compared with the international nabP+gemcitabine registration MPACT trial,^2^ the SIEGE trial patient demographics were very similar. The ORR in the CON arm of SIEGE (30%) and in MPACT (29% by an investigator and 23% by an independent review) was almost identical, although median PFS and OS in the CON arm of SIEGE (4.3 and 7.9 months) were shorter than in MPACT (5.5 and 8.5 months). Measuring objective response and therefore PFS is notably difficult in PDAC, so OS is recognised as a much more robust endpoint. Although a trend for OS gain was seen, this lacked statistical significance in this non-selected patient population, calling into question the overall clinical applicability of this novel schedule. However, this relatively small randomised study was not powered to show a difference in OS.

Reassuringly, despite additional hospital visit requirements, we found no evidence that SEQ delivery generated higher levels of non-compliance or patient withdrawals. Furthermore, patient safety and QoL did not appear to be compromised. SEQ therapy was associated with more treatment-related AEs, with twice the incidence of grade ≥3 neutropaenia and use of secondary GCSF prophylaxis, but these did not translate into loss of patient QoL compared with CON delivery. In part, this is likely to reflect the nature of AEs occurring, some of which were changes in blood parameters unlikely to have immediate sequelae for patients. Other common toxicities associated with nabP+gemcitabine did not appear to be significantly influenced by treatment schedule.

The QoL data presented here is the most comprehensive analysis of patient experience receiving standard CON delivery of nabP+gemcitabine undertaken to date, which also enabled comparison with the experimental SEQ regimen. In the MPACT registration trial, QoL was indirectly measured using the Quality-Adjusted Time Without Symptoms (Q-TWIST) to compare QoL and survival associated with nabP+gemcitabine with gemcitabine alone. Relative gains in Q-TWIST favoured the combination regimen, ranging from 12 to 30%.^12^ Limited data from a phase II study evaluating QoL with the EORTC QLQ-C30 instrument in 146 locally advanced and metastatic PDAC patients randomised to receive either nabP+gemcitabine or gemcitabine alone have been reported in abstract form only to date, but indicated that QoL scores were improved and sustained with the combination compared with gemcitabine alone.^13^ Our findings support these preliminary data and go further to provide more detailed insight into the patient experience with nabP+gemcitabine chemotherapy. Some key findings from the patient questionnaires reported here are of particular importance. Firstly, mean pre-treatment QLQ-C30 global health scores were low, reflecting overall poor QoL associated with a diagnosis of advanced PDAC.^14^ Global health status scores were remarkably stable throughout treatment, while the fall in QoL in the CON arm seen at 6 months might be consistent with higher rates of patients progressing in this arm by this time point. Secondly, the initial months of treatment were associated with progressive loss of physical, social and role function for patients on both arms of the trial, which persisted over time, suggesting that chemotherapy confers considerable interference with patients’ activities of daily living. Thirdly, the QoL questionnaires picked out evidence of improvement in disease-related symptoms while on treatment, in particular pain, pancreatic pain, appetite and insomnia. These symptoms have been previously identified as being particularly problematic for patients with PDAC,^15^ suggesting that these gains may be clinically relevant for this patient group. Other side effects known to be associated with nabP+gemcitabine chemotherapy administration were more apparent while on treatment, in particular, breathlessness and altered bowel habit, although these were less problematic with SEQ compared with CON delivery.

Our clinical findings support the animal model predicting improved efficacy of nabP+gemcitabine by exposing the cancer to nabP in advance of gemcitabine delivery, lending weight to the theory that nabP enhances gemcitabine cytotoxicity.^6,7^ The gemcitabine-metabolising enzyme, CDA, has been implicated in influencing patient response to treatment with gemcitabine.^14,16–18^ CDA is prone to genetic polymorphism, and some mutations have been reported to influence gemcitabine exposure levels and related toxicities.^19,20^ Genotyping CDA as a marker of gemcitabine response and toxicity has been explored, while CDA functional testing offers a simpler, easier methodology.^14–16^ However, after adjustment for neutrophil count, we could not find any correlation between whole-blood CDA activity and toxicity, or efficacy associated with nabP+gemcitabine. Our findings reinforce those reported in the only other prospective study evaluating CDA activity in a cohort of 120 patients with resected PDAC who received adjuvant gemcitabine monotherapy.^17^ In that study, CDA activity was measured in serum,^14–17^ which explains the difference in the range of CDA activity levels we have reported.^21,22^ The reduction in both unadjusted and adjusted CDA activity measured prior to day 8, cycle 1 in the SEQ arm could reflect an early signal of altered gemcitabine metabolism associated with the enhanced activity of SEQ administration.

Immunohistochemical studies showed that CDA staining was identified primarily but not exclusively in the cellular compartment of tumours, but only strongly positive CDA staining within the tumour epithelial cells predicted for survival benefit with SEQ delivery. Strong CDA expression in one-third of tumours tested suggests that further evaluation of this potential novel biomarker in future PDAC interventional trials may be of value.

Given the alternative hypothesis that nabP influences the tumour stroma, we quantified stroma content in the pre-treatment tumour biopsies. Moderate–extensive stromal content predicted for a better outcome following nabP+gemcitabine chemotherapy, irrespective of trial arms. PDAC is histologically characterised by a dense stroma, which may promote tumour growth, invasion and resistance to anticancer drug therapy,^23^ although recent research suggests that some elements of the stroma may actually restrain tumour growth.^24^ Initial studies suggested that nabP may exert its effect by inhibiting the stromal protein, SPARC,^2^ but retrospective assessment of tumour tissue collected from patients recruited to the MPACT trial did not demonstrate any correlation between tumour SPARC expression and treatment outcome.^25^ Genetically engineered mouse models lacking SPARC did not result in decreased intra-tumoural nabP concentrations or treatment efficacy,^7^ further placing the role of SPARC in nabP efficacy in doubt. A better understanding of how therapeutic interventions impact complex cancer cell–tumour stroma interactions is clearly needed.

This feasibility trial has important limitations: it was underpowered to detect an OS difference, while the primary endpoint, PFS, was assessed by local investigator review, with limited central review. Therefore, this study can only be considered hypothesis-generating. Even so, tumour expression of key proteins implicated in the mechanism of action of nabP+gemcitabine is of interest: patients with more extensive tumour stroma content had longer survival, irrespective of drug scheduling and strong intra-tumoural CDA expression predicted for benefit from SEQ administration of nabP+gemcitabine. These novel findings offer opportunities for further exploration in future biomarker-driven, prospective trials undertaken in patients with PDAC.

## Supplementary information

Supplementary Tables and Figures

## Data Availability

The SIEGE trial data is held by the Cambridge Clinical Trials Unit—Cancer Theme. Ownership of the data resides with the Trial Management Group (TMG). Access to the data can be requested and authorised by the TMG.
