# Supplementary material for: Scheduling nab-paclitaxel combined with gemcitabine as first-line treatment for metastatic pancreatic adenocarcinoma
Source: Br J Cancer. 2020 Apr 30;122(12):1760–8. doi: 10.1038/s41416-020-0846-2 (PMC7283477; doi:10.1038/s41416-020-0846-2)
Supplement: Supplementary file 1 — Supplementary Tables and Figures [file 41416_2020_846_MOESM1_ESM.pdf]

Supplementary Information

Scheduling nab-Paclitaxel combined with gemcitabine as first line treatment for metastatic pancreatic adenocarcinoma

Supplementary Tables

Table S1: RECIST v1.1 objective response*

**Best response**	**Concomitant (N=75)**	**Sequential (N=71)**
Complete response (CR)	0	1 (2%)
Partial response (PR)	19 (31%)	28 (50%)
Stable disease	29 (48%)	20 (36%)
Progressive disease	13 (21%)	7 (13%)
Total no. assessed for response	61	56
Overall response rate (CR+PR)<br>p=0.023	31%(95%CI 20%-44%)	52%(95%CI 38%-65%)
No. patients not assessable for response	14	15
No. of cycles received for patients with no response assessment:		
0	1	3
1	10	11
2	3	1

*Response was evaluated by investigators and central radiological review was performed for 10% of randomly selected patients with good concordance. Most discrepancies related to non-target lesions and did not affect key outcome measures.

Table S2: Summary of survival outcomes

		Concomitant	Sequential
All patients	N	75	71
PFS	Median (months) (95%CI)	4.0 (3.0 – 5.4)	5.6 (3.6 – 7.2)
	6 month (95%CI)	32% (21% - 43%)	46% (34% - 58%)
		HR=0.67, CI=0.47- 0.95, p log-rank=0.022	
OS	Median (months) (95%CI)	8.2 (6.1 – 10.0)	10.2 (6.3 – 11.5)
	1 year (95%CI)	28% (18% - 38%)	29% (18% - 40%)
		HR=0.93, CI=0.65-1.33, p log-rank=0.70	
Evaluable patients*	N	48	50
PFS	Median (months) (95%CI)	5.2 (3.8 – 6.1)	7.2 (5.5 – 8.8)
	6 month (95%CI)	40% (26% - 53%)	57% (43% - 71%)
		HR=0.60, CI=0.40-0.90, p log-rank= 0.014	
OS	Median (months) (95%CI)	9.7 (7.4 – 11.6)	11.2 (10.1 – 11.9)
	1year (95%CI)	31% (18% - 44%)	36% (23% - 49%)
		HR=0.83, CI=0.54-1.28, p log-rank= 0.40	

*patients who completed at least 2 cycles of planned treatment and at least 2 of the 3 planned treatment days with both drugs administered in each cycle

Table S3: EORTC QLQ-C30 and PAN26 QoL questionnaire scores – changes from baseline (pretreatment) at week 12 and week 26, by trial arm. Clinically important differences are considered small for mean score changes of 5-10 points, moderate for 10-20 points and large for >20 points. Moderate/large gains in QOL scores occurring in both arms in at least 1 time point are highlighted in green. Moderate/large losses in QoL scores occurring in both arms in at least 1 time point are highlighted in pink. Moderate/large gains in QoL scores occurring in one arm only in at least 1 time point are highlighted in yellow. Moderate/large losses in QoL scores occurring in one arm only in at least 1 time point are highlighted in orange.
95%CI and p values for the difference between arms are provided in parentheses.

		Baseline	Week 12	Week 26
	**EORTC QLQ –C30**			
Global health status / QoL	Concomitant	63.16(19.82)	2.95	-7.49
	Sequential	60.61(21.41)	-3.73	-0.54
	Difference between arms		6.68(-2.07-15.43;0.132)	-6.95(-15.96-2.06;0.128)
Physical Functioning	Concomitant	76.23(20.52)	-10.89	-13.89
	Sequential	71.54(24.07)	-11.26	-10.11
	Difference between arms		0.37(-8.9-9.65;0.936)	-3.77(-13.47-5.92;0.439)
Role Functioning	Concomitant	65.46(32.25)	-11.40	-13.86
	Sequential	64.46(31.00)	-10.42	-15.95
	Difference between arms		-0.98(-14.71-12.76;0.888)	2.09(-13.02-17.2;0.783)
Emotional Functioning	Concomitant	74.40(22.06)	7.02	5.41
	Sequential	71.09(25.66)	6.60	4.93
	Difference between arms		0.41(-7.96-8.79;0.922)	0.48(-7.8-8.75;0.908)
Cognitive Functioning	Concomitant	81.43(23.15)	-2.78	-2.27
	Sequential	79.80(24.02)	-0.65	2.04
	Difference between arms		-2.13(-10-5.73;0.590)	-4.31(-12.18-3.55;0.276)
Social Functioning	Concomitant	73.43(27.01)	-8.07	-10.74
	Sequential	67.93(30.98)	-7.44	-12.16
	Difference between arms		-0.63(-13.19-11.93;0.921)	1.42(-11.13-13.97;0.821)
Fatigue	Concomitant	40.90(25.74)	7.51	7.76
	Sequential	43.06(27.02)	5.13	7.19
	Difference between arms		2.39(-9.45-14.22;0.689)	0.56(-10.97-12.09;0.922)
Nausea / Vomiting	Concomitant	14.25(20.27)	1.83	-4.38
	Sequential	15.69(21.72)	-2.47	0.71
	Difference between arms		4.3(-5.87-14.48;0.402)	-5.09(-13.33-3.15;0.221)
Pain	Concomitant	38.10(27.97)	-10.91	0.23
	Sequential	39.22(31.38)	-14.81	-5.86
	Difference between arms		3.9(-8.59-16.4;0.535)	6.09(-8.89-21.07;0.419)
Dyspnoea	Concomitant	14.93(24.12)	9.06	16.73
	Sequential	20.40(28.99)	8.68	3.81
	Difference between arms		0.38(-13.28-14.05;0.956)	12.92(-0.81-26.65;0.065)
Insomnia	Concomitant	39.13(31.81)	-17.45	-12.50
	Sequential	37.81(31.73)	-17.04	-14.21
	Difference between arms		-0.42(-12.51-11.68;0.945)	1.71(-12.26-15.69;0.807)
Appetite loss	Concomitant	42.16(36.70)	-0.57	-15.42
	Sequential	50.00(35.73)	-14.73	-9.48

		Baseline	Week 12	Week 26
	Difference between arms		14.17(0.2-28.13;0.047)	-5.93(-21.11-9.24;0.437)
Constipation	Concomitant	31.86(30.71)	-11.97	-8.93
	Sequential	29.41(35.28)	-14.95	-7.93
	Difference between arms		2.98(-9.24-15.2;0.628)	-1(-15.12-13.13;0.888)
Diarrhoea	Concomitant	15.24(24.53)	6.36	1.72
	Sequential	18.18(29.92)	-0.50	-1.21
	Difference between arms		6.86(-4.66-18.37;0.239)	2.93(-9.91-15.77;0.650)
Financial Problems	Concomitant	14.49(25.87)	-6.16	-2.58
	Sequential	31.28(36.74)	2.97	1.00
	Difference between arms		-9.13(-20.27-2.02;0.107)	-3.59(-13.62-6.44;0.477)
	**EORTC QLQ-PAN26**			
Pancreatic pain	Concomitant	37.56(19.42)	-16.66	-7.85
	Sequential	41.42(24.71)	-20.46	-15.71
	Difference between arms		3.79(-5.51-13.09;0.419)	7.86(-4.09-19.8;0.192)
Digestive	Concomitant	32.85(31.57)	-3.88	-11.56
	Sequential	43.28(34.09)	-7.73	-4.32
	Difference between arms		3.85(-8.98-16.68;0.551)	-7.24(-21.7-7.22;0.320)
Altered bowel habit	Concomitant	29.10(23.09)	12.28	13.18
	Sequential	28.61(28.55)	4.35	-3.42
	Difference between arms		7.93(-2.66-18.52;0.140)	16.6(5.94-27.26;0.003)
Hepatic	Concomitant	7.25(11.95)	-3.39	-2.69
	Sequential	13.93(23.32)	-4.61	-0.38
	Difference between arms		1.23(-4.45-6.9;0.668)	-2.31(-10.85-6.23;0.590)
Body image	Concomitant	24.15(28.08)	12.78	7.67
	Sequential	26.62(30.02)	7.76	6.44
	Difference between arms		5.02(-7.55-17.59;0.429)	1.22(-10.65-13.09;0.837)
Health care satisfaction	Concomitant	85.07(21.54)	-9.30	-4.65
	Sequential	86.36(20.45)	-0.42	2.45
	Difference between arms		-8.88(-18.78-1.02;0.078)	-7.1(-16.6-2.4;0.140)
Sexuality	Concomitant	56.11(41.96)	8.83	3.50
	Sequential	51.44(37.24)	17.49	8.89
	Difference between arms		-8.65(-24.47-7.17;0.278)	-5.38(-24.17-13.4;0.566)

Table S4. Correlation between pre-treatment whole blood CDA activity and patient characteristics

Patient characteristics	Univariable			multivariable regression
	Pearson Correlation coefficient	Spearman Correlation coefficients	Wilcoxon/Kruskal-Wallis test	p value
Age	-0.068, p=0.44	-0.074, p=0.40		0.75
Gender			P=0.83	0.14
Karnofsky PS	-0.23, p=0.0061	-0.20, p=0.019	P=0.042	0.21
Site of primary tumour			P=0.30	0.80
Liver metastases			P=0.10	0.93
CA19.9 (U/ml)	0.076, p=0.40	0.13, p=0.13		0.71
C-Reactive protein(mg/L)	0.54, p<0.0001	0.35, p<0.0001		0.30
Neutrophil count (10^9/L)	0.82, p<0.0001	0.82, p<0.0001		<0.0001

Table S5. Pre-treatment whole blood CDA activity as a continuous variable on PFS and OS

	HR (95%CI), p value in Cox regression model	
	Unadjusted (univariate)	Adjusted for baseline neutrophil count
PFS	1.03 (1.01-1.06), p=0.0012	1.00 (0.97-1.04), p=0.78
OS	1.05 (1.02-1.07), p<0.0001	1.02 (0.99-1.06), p=0.24

Table S6. Whole blood CDA activity, by tumour epithelial CDA expression

Tissue tumour epithelial CDA expression	N	Mean	Median	Std Dev	25th Pctl	75th Pctl	Minimum	Maximum
Negative/weakly positive/positive	67	14.10	10.33	8.77	8.47	17.55	4.14	43.85
Strongly positive	33	16.07	13.70	7.28	12.05	19.15	6.88	35.60
Unknown	32	12.53	11.90	7.28	7.96	15.35	3.75	41.50

P value of Wilcoxon test =0.031

Supplementary Figures

Figure S1 Consort Diagram

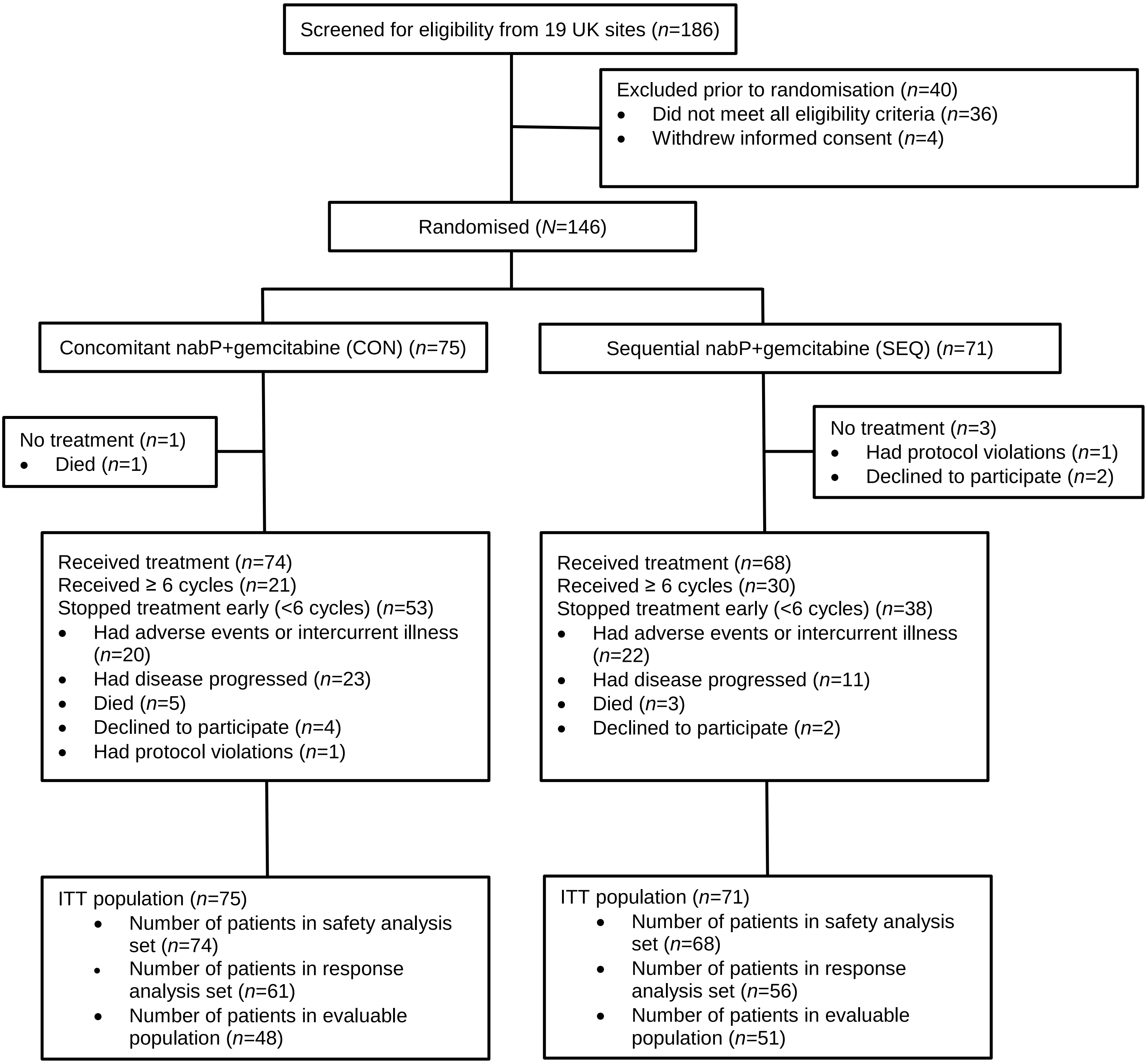

Figure S2. Scatter plot demonstrating correlation between pre-treatment CDA activity and neutrophil count

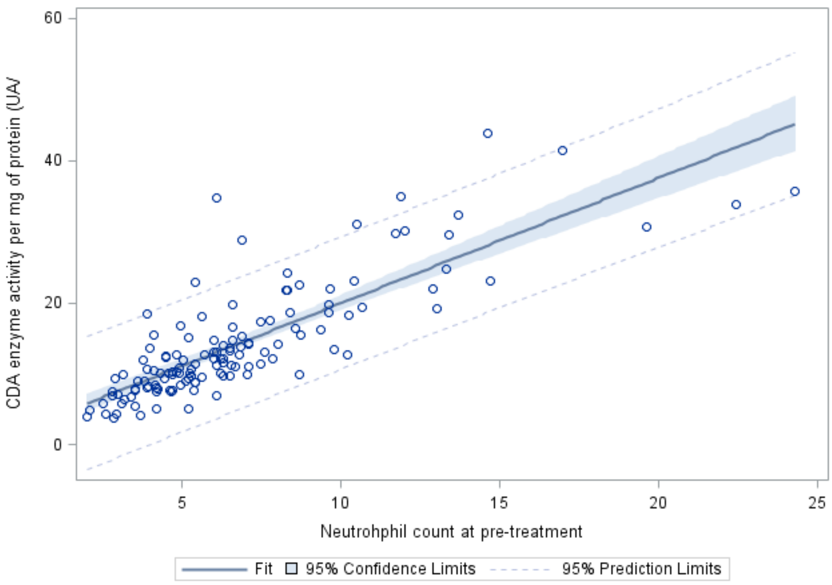

Figure S3: Box plot for pretreatment whole blood CDA activity and any haematological CTCAE grade during (A) cycle 1 and (B) during first 2 cycles of chemotherapy (both arms combined)

(A)

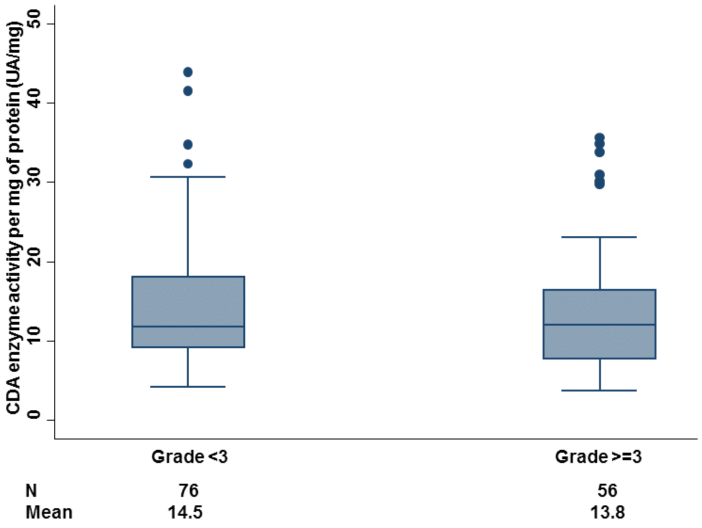

(B)

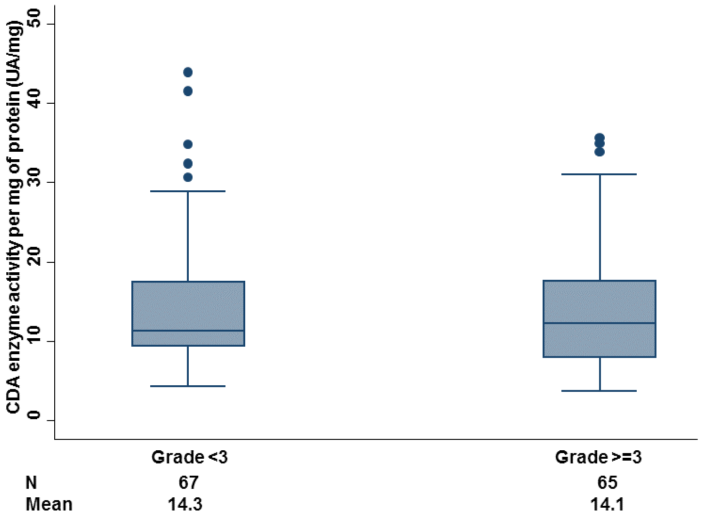

Figure S4. Median whole blood CDA activity measured from start of cycle 1 until start of cycle 2 chemotherapy, by trial arm

a) Unadjusted CDA activity

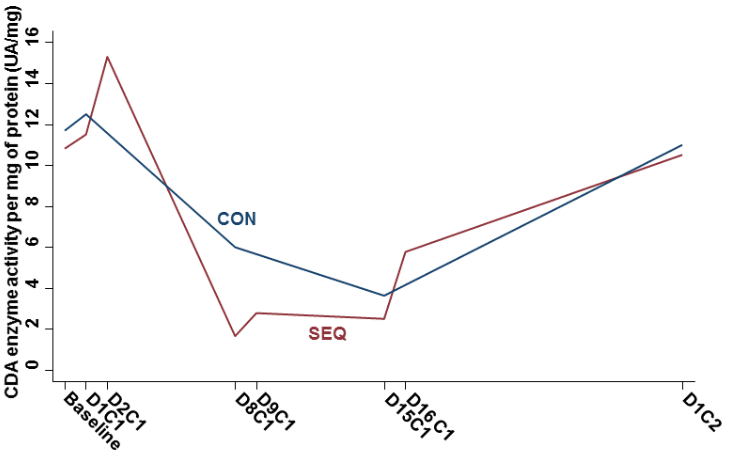

b) CDA activity, after adjusting for neutrophil count (CDA/neutrophil count)

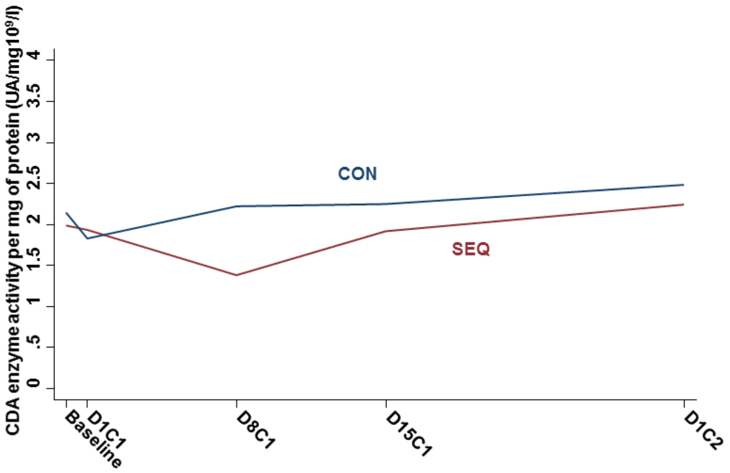

Figure S5. Kaplan-Meier plots of PFS and OS, by subgroups determined by pretreatment tumour biopsy immunohistochemical analyses

A) Tumour grade

	All patients by tumour grade level<br>p of log-rank	Well/moderately differentiated by arm<br>HR(95%CI)	Poorly differentiated by arm<br>HR(95%CI)
**PFS**	p =0.41<br>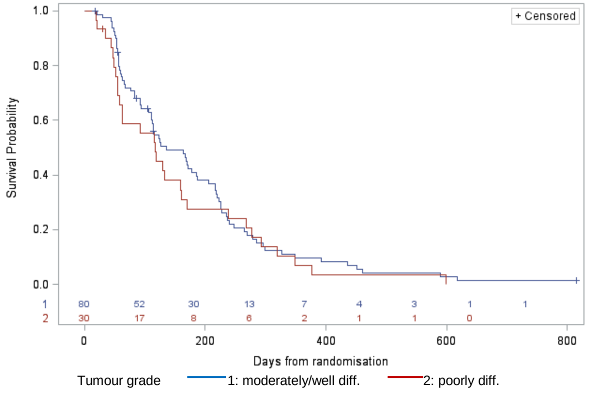 	0.65 (0.41-1.04)<br>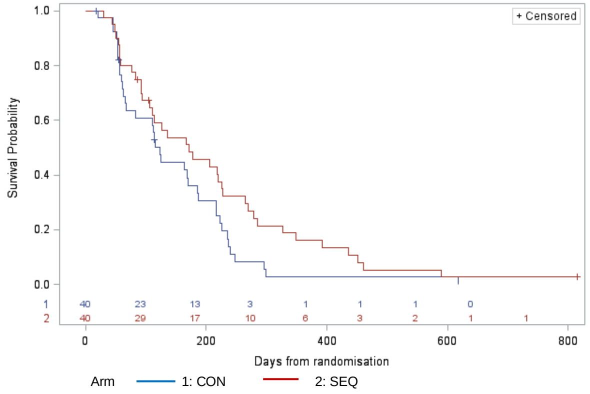 	0.77 (0.35-1.70)<br>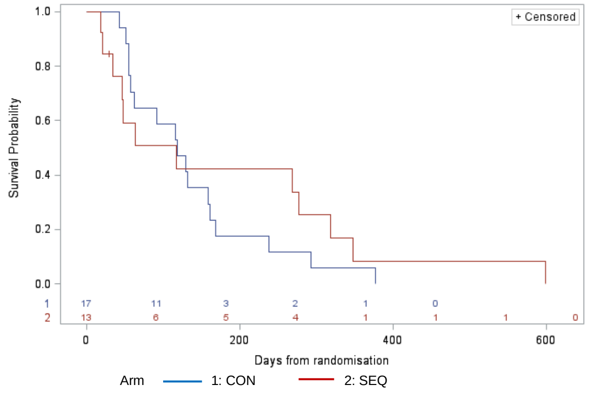
**OS**	p =0.12<br>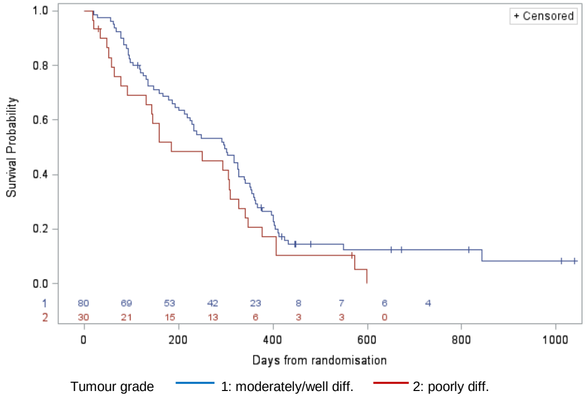 	0.86 (0.53-1.39)<br>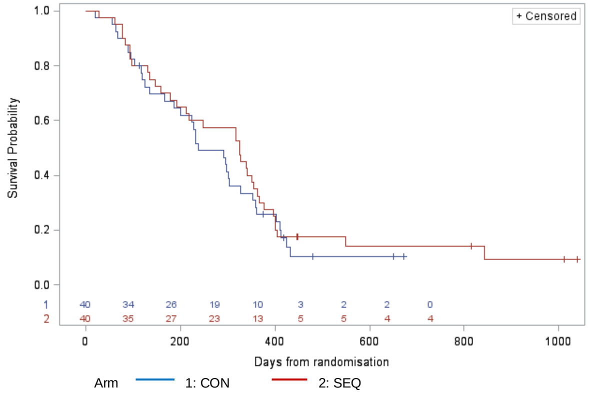 	1.08 (0.50-2.33)<br>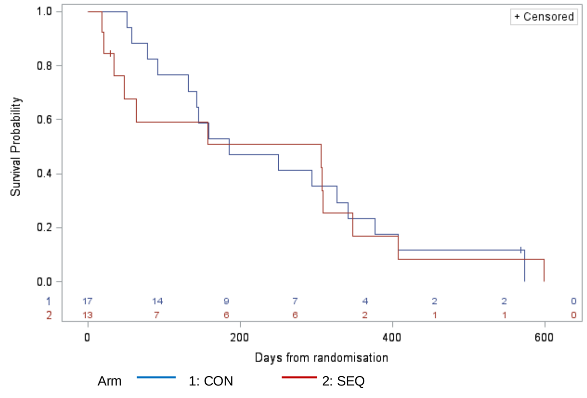

B) CDA expression – Tumour epithelium

	All patients, by CDA expression<br>p –value of log-rank test	Negative/weakly positive/positive, by trial arm<br>HR (95%CI)	Strongly positive, by trial arm<br>HR (95%CI)
**PFS**	p =0.24<br>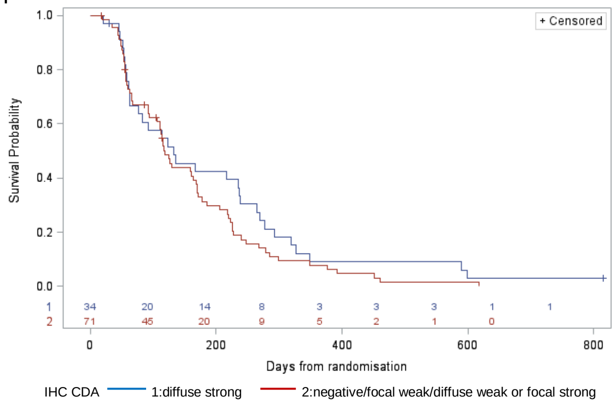	0.91 (0.56-1.48)<br>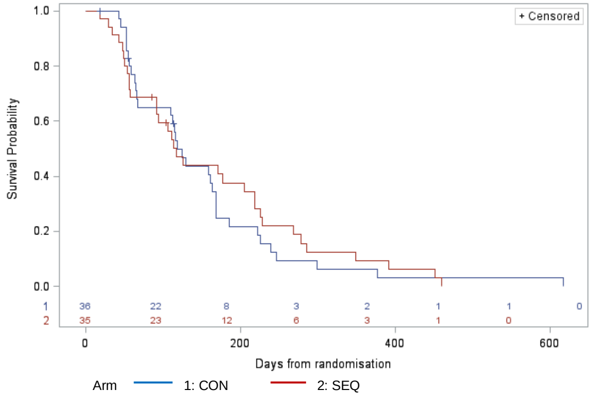	**0.31 (0.13-0.70)**<br>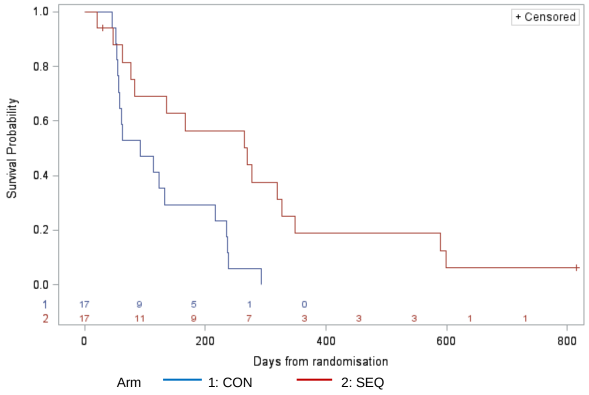
**OS**	p=0.88<br>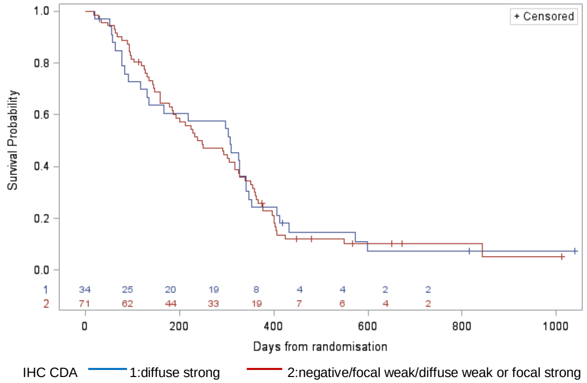	1.07 (0.65-1.76)<br>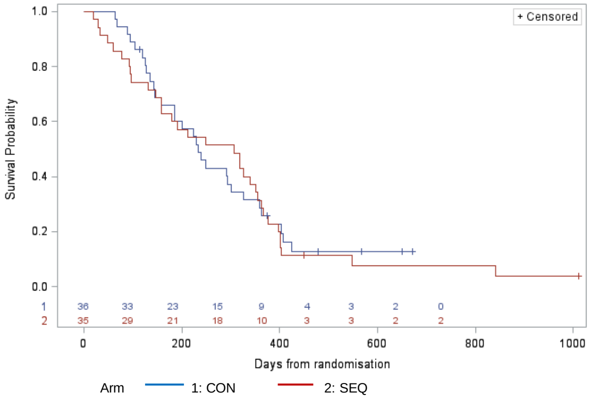	0.740.36-1.56)<br>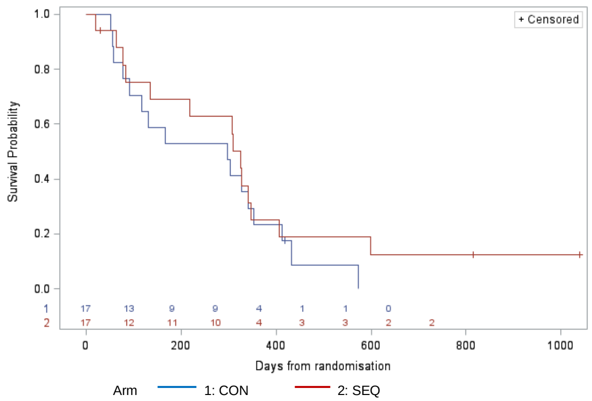

C) CDA expression – stromal cells

<table>
<tr><th></th><th>All patients, by CDA expression
p –value of log-rank test</th><th>Weakly positive/positive, by trial arm
HR (95%CI)</th><th>Strongly positive, by trial arm
HR (95%CI)</th></tr>
<tr><td>PFS</td><td>p=0.12
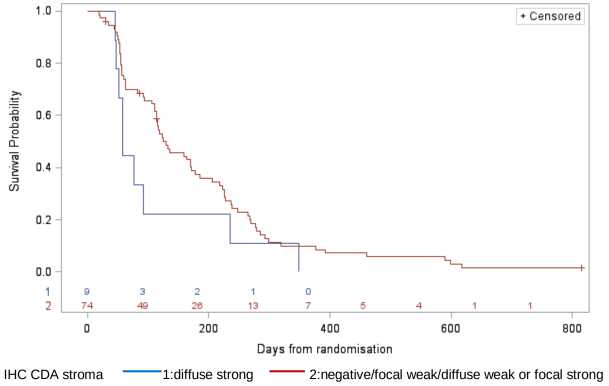
</td><td>0.74 (0.46-1.20)
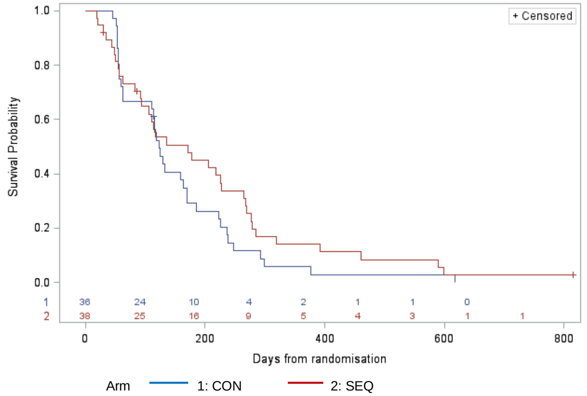
</td><td>0.73 (0.17-3.07)
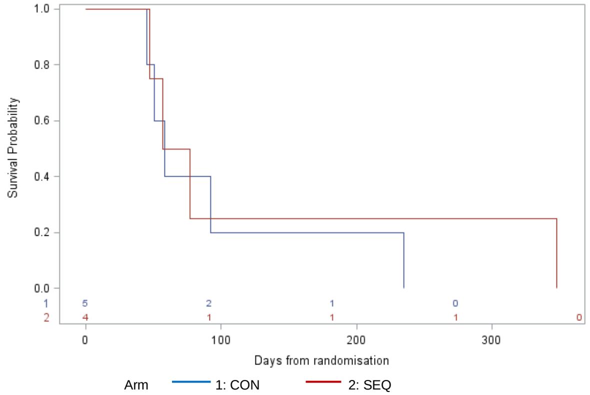
</td></tr>
<tr><td>OS</td><td>p=0.48
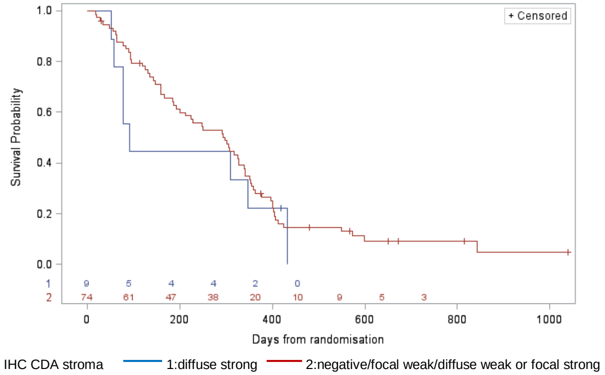
</td><td>1.10 (0.67-1.79)
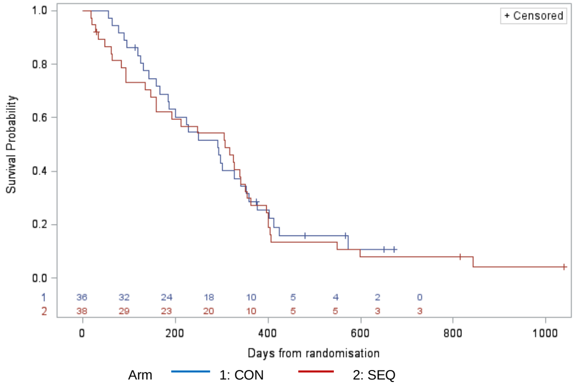
</td><td>1.490.33-6.81)
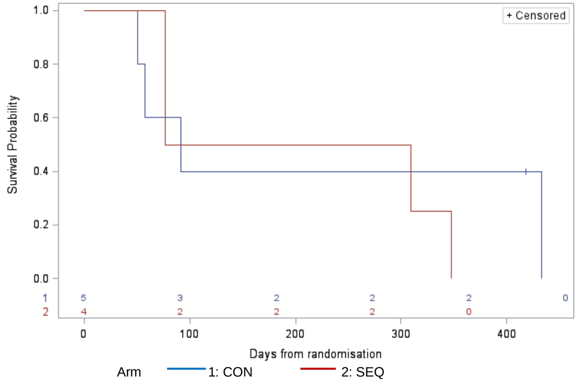
</td></tr>
</table>

D) Presence of tumour stroma

<table>
<tr><td></td><td>All patients, by presence of tumour stroma
p –value of log-rank test</td><td>No/minimal stroma, by trial arm
HR (95%CI)</td><td>Moderate/extensive stroma, by trial arm
HR (95%CI)</td></tr>
<tr><td>PFS</td><td>p =0.027

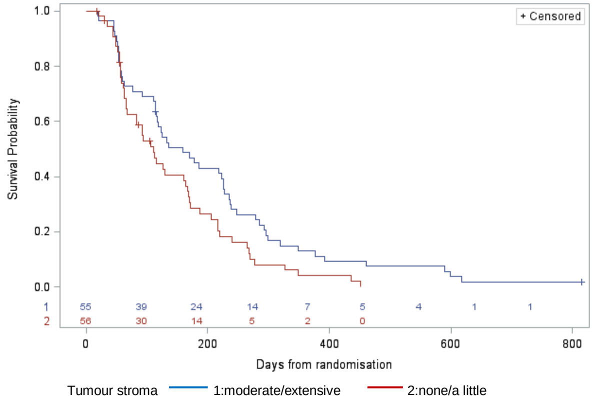

</td><td>0.56 (0.30-1.03)

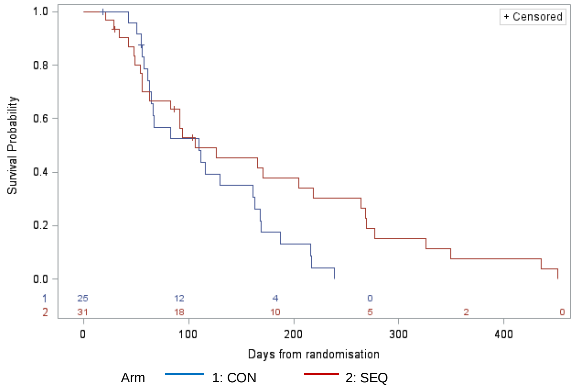

</td><td>0.60 (0.34-1.06)

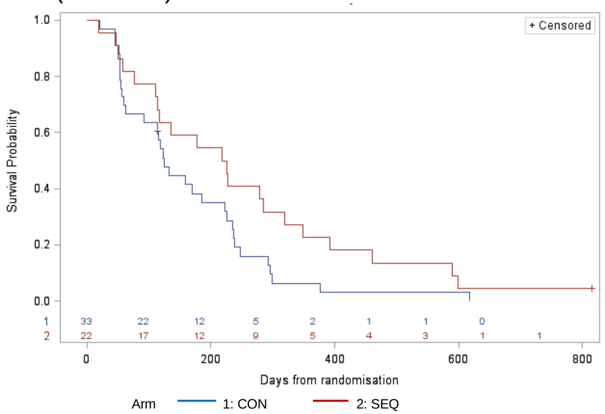

</td></tr>
<tr><td>OS</td><td>p=0.023

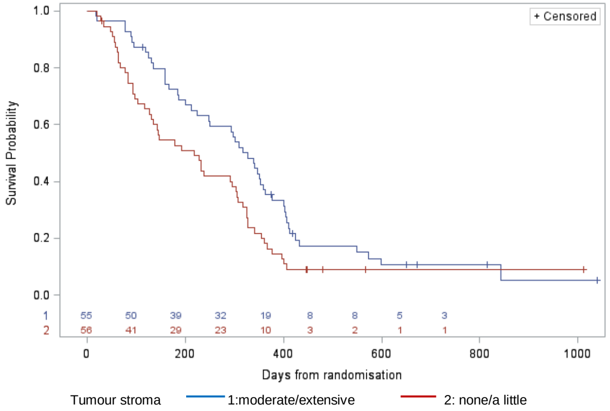

</td><td>0.79 (0.45-1.38)

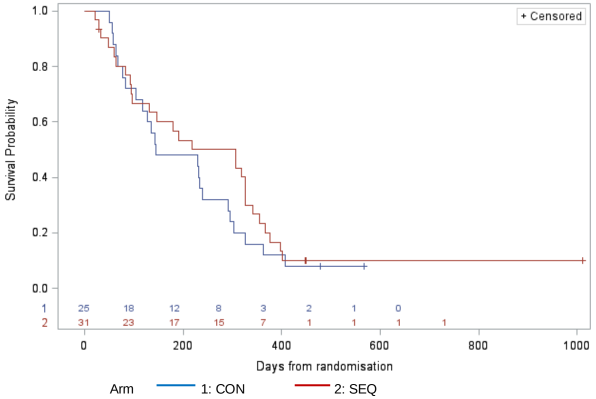

</td><td>0.85 (0.47-1.53)

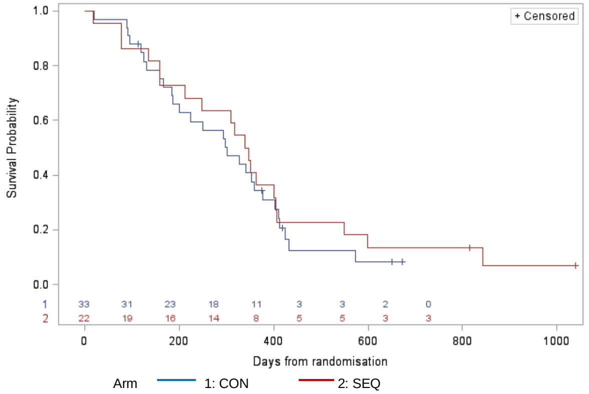

</td></tr>
</table>
